# Early dynamics of *Toxoplasma gondii* infection in sheep inoculated at mid-gestation with archetypal type II oocysts

**DOI:** 10.1186/s13567-025-01557-1

**Published:** 2025-07-01

**Authors:** Roberto Sánchez-Sánchez, Pilar Horcajo, Miguel Fernández, David Arranz-Solís, Natalia Velasco-Jiménez, Michela Re, Daniel Gutiérrez-Expósito, Guillermo Valdivia, Angela Alonso-Diez, Julio Benavides, Luis Miguel Ortega-Mora

**Affiliations:** 1 SALUVET, Animal Health Department, Faculty of Veterinary Sciences, Complutense University of Madrid, Ciudad Universitaria S/N, 28040 Madrid, Spain; 2 Animal Health Department, University of Leon, Campus de Vegazana S/N, 24071 León, Spain; 3 Animal Medicine and Surgery Department, Faculty of Veterinary Sciences, Complutense University of Madrid, Ciudad Universitaria S/N, 28040 Madrid, Spain; 4 Instituto de Ganadería de Montaña (CSIC-Universidad de León), León, Spain

**Keywords:** *Toxoplasmagondii*, sheep, mid-gestation, early abortion, early infection dynamics, small intestine, mesenteric lymph nodes, placental thrombosis

## Abstract

Early abortion is a clinical presentation of ovine toxoplasmosis that occurs in the second week post-infection (pi), which is characterised by placental infarcts, foetal leukomalacia and absence of parasites in the placenta and foetal tissues. The pathogenic mechanism of early abortion is unknown*,* and descriptions of the early dynamics of *T. gondii* infection in pregnant sheep are scarce. The aim of this study was to investigate the presence of lesions and parasite DNA in the small intestine, mesenteric lymph nodes and placenta/foetus, that could be key during the first week after oral infection in sheep at mid-pregnancy. In the small intestine, lesions were rare and parasite DNA detection rates were low (3–8%), with the highest parasite DNA detection and burden found on day 6 pi in the Peyer’s patches of the medial jejunum. In the mesenteric lymph nodes, adenomegaly and microscopic lesions were mainly observed on day 6 pi. Parasite DNA was detected in 11% and 61.2% of the samples from mesenteric lymph nodes on days 3 and 6 pi, respectively, with higher parasite DNA detection rates and burdens in the medial and distal jejunal lymph nodes on day 6 pi. In the placentomes, on day 6 pi, gross lesions were not observed, although significant histological changes, such as endothelial activation and vascular thrombosis, were found in 18.6% and 8.3% of the placentomes, respectively. These findings lay the groundwork for future research aimed at elucidating the precise mechanisms underlying early abortions following *T. gondii* infection in pregnant sheep.

**Supplementary Information:**

The online version contains supplementary material available at 10.1186/s13567-025-01557-1.

## Introduction

*Toxoplasma gondii* is a widespread zoonotic apicomplexan parasite able to infect any warm-blooded animal. In veterinary medicine, *T. gondii* infection during pregnancy may result in foetal loss and stillbirths in some relevant domestic species, such as sheep and goats [1]. In fact, abortions caused by *T. gondii* represent 10–23% of ovine abortions in Europe and the USA and 3–54% in the Middle East and South America, causing important economic losses [2, 3].

In sheep, oral ingestion of contaminated food or water containing sporulated oocysts initiates the infection process. Oocysts excyst in the small intestine, releasing sporozoites that invade enterocytes, transform into tachyzoites, and cross the intestinal barrier. During the first week post-infection (pi), tachyzoites replicate locally in intestinal tissues and mesenteric lymph nodes before being disseminated via the bloodstream [4]. In pregnant sheep, tachyzoites are detected in the placenta around day 14 pi, where they replicate and reach the foetus [5]. However, after 14 days pi, the presence of tachyzoites is suppressed by the host immune response, and bradyzoites (within tissue cysts), which are less affected by this immune response, become the dominant parasitic stage, establishing the chronic stage of infection [6].

The outcome of *T. gondii* infection in pregnant sheep is highly dependent on the gestational stage at which infection occurs: infection during the first two-thirds of pregnancy usually results in resorption or abortion, whereas infection during late pregnancy usually results in stillbirth or birth of weak lambs due to partial control of parasite replication by the developing foetal immune system [7, 8]. In any case, two clinical presentations of toxoplasmosis-related abortions have been described in sheep, distinguished by the timing post-infection (dpi) when abortions occur: early abortion (7–14 dpi) and late abortion (28 dpi onwards). Early abortions have been described in experimental infections in sheep [9–16], but also in goats [17], and might be underdiagnosed in the field, as, unlike late abortions, early abortions often lack detectable parasites in the placenta or foetal tissues, as well as *T. gondii*-specific IgG antibodies in the dam at the time of abortion. Moreover, histological lesions in the placenta differ between both clinical presentations: while early abortions are characterised by thrombotic lesions and infarcts in placentomes, late abortions are characterized by multifocal necrosis [18]. Several investigations in the last decade have shown that early abortions may occur after sheep are infected at different stages of gestation [8] with different *T. gondii* genotypes [5] and even after infection with as few as 10 sporulated oocysts [19]. The pathogenic mechanism underlying early abortion remains unclear, although it is hypothesised that placental thrombosis leading to early abortion results from an exacerbated proinflammatory immune response triggered in tissues involved in the early dynamics of infection, such as the small intestine and mesenteric lymph nodes, following parasite replication during acute infection [18].

Most research on the early dynamics and immune response to *T. gondii* infection has been conducted in murine models [20–24]. However, anatomical and physiological differences between mice and sheep, particularly in the digestive tract [25–27] and the immune response during *T. gondii* infection [28], limit the applicability of findings from murine studies to sheep. Studies on the early dynamics of *T. gondii* infection in sheep are scarce and have focused on acquired toxoplasmosis in lambs [4, 9] or pregnant sheep [16]. Therefore, the aim of our study was to investigate the early dynamics of *T. gondii* infection in sheep infected with type II (genotype #3) oocysts at mid-pregnancy by examining parasite presence and lesions in the small intestine, mesenteric lymph nodes, placenta and foetus during the first week of infection.

## Materials and methods

### Ethics statement

This experiment involving animals was authorized by the Animal Welfare Committee of the Community of Madrid, Spain (PROEX 68.0/20), following procedures described in the Spanish and European Union legal requirements (Law 32/2007, R.D. 53/2013, and Council Directive 2010/63/EU). Good clinical practices were implemented to guarantee animal welfare.

### Animals and experimental design

Twenty-one (*n* = 21) pure Rasa Aragonesa breed pregnant sheep, aged 18 months, were selected from a commercial flock. All the animals were seronegative for *T. gondii*, *Neospora caninum*, border disease virus (BDV), Schmallenberg virus (SBV), *Coxiella burnetii*, and *Chlamydia abortus*, as determined by ELISA. During reproductive management, they were oestrus synchronized and mated with pure-bred Rasa Aragonesa rams for 2 days. Pregnancy was confirmed by ultrasound scanning on day 40 post mating.

**Figure 1:**
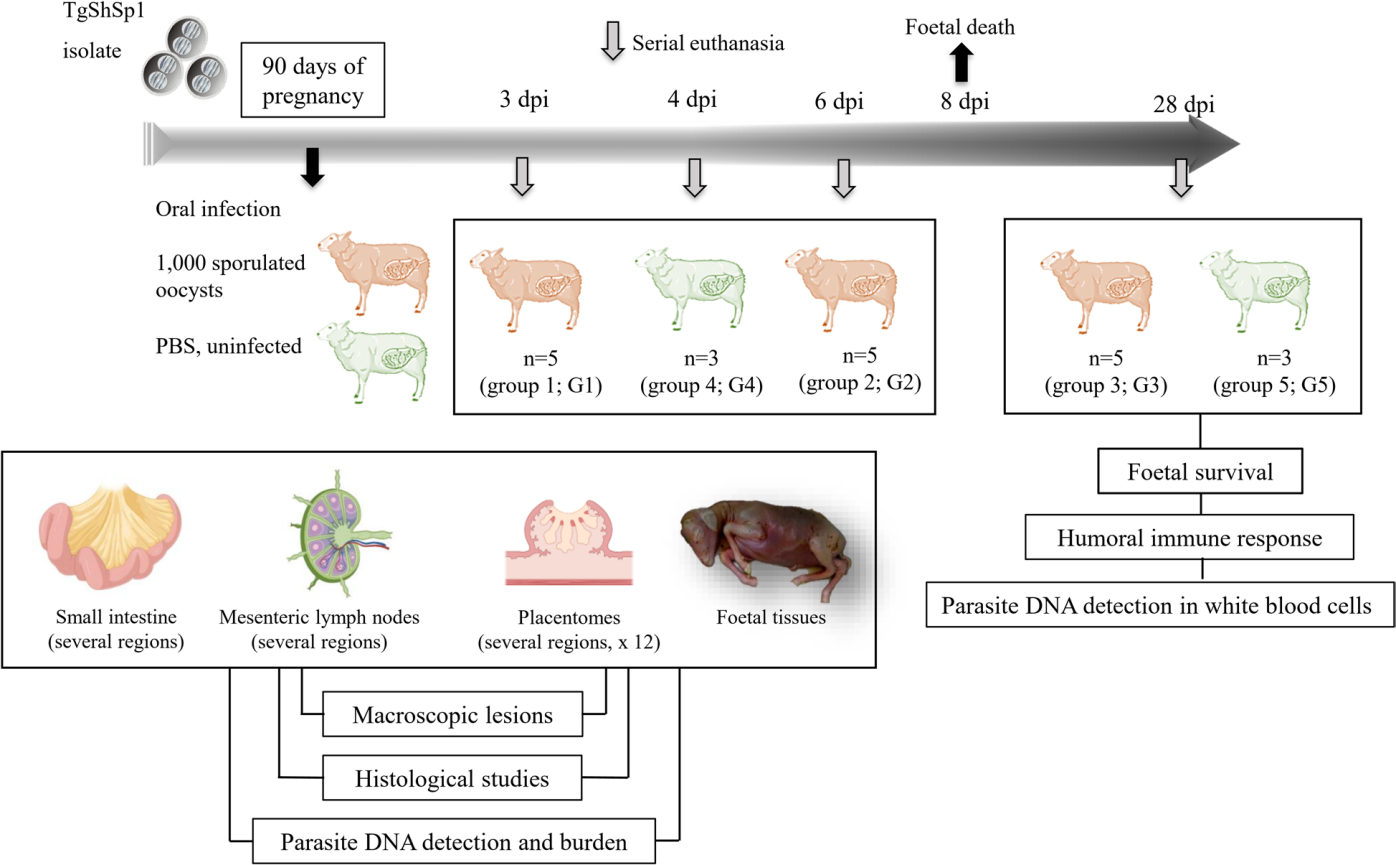
**Overview of the experimental design. dpi: days post-infection**.

Pregnant sheep were randomly distributed into five experimental groups and housed at the animal facilities of the Animal Health Department at the Faculty of Veterinary Sciences (Complutense University of Madrid, Spain). Sheep had access to water ad libitum and were fed barley straw ad libitum as fodder and two daily rations of pelleted feed. Fifteen sheep were allocated into groups 1 (G1; *n* = 5), 2 (G2; *n* = 5) and 3 (G3; *n* = 5), which were dosed orally with 1000 *T. gondii* sporulated oocysts of the TgShSp1 isolate (PCR–RFLP genotype #3) on day 90 of gestation (dg) [19]. The oocysts were shed by a cat after oral infection with brains from chronically infected mice, sporulated for 7 days at room temperature and stored at 4 °C for one year until use [19]. The six remaining pregnant sheep were allocated into uninfected groups 4 (G4; *n* = 3) and 5 (G5; *n* = 3), which received 8 mL of phosphate-buffered saline (PBS) at 90 dg (Figure 1).

Infected sheep from G1 and G2 were euthanized on days 3 and 6 pi, respectively (before the occurrence of early abortions), and infected sheep from G3 were maintained until day 28 pi. Uninfected sheep from G4 and G5 were euthanized on days 4 and 28 pi, respectively (Figure 1) and served as non-infected controls. Sheep were sedated with 0.1 mg/kg of xylazine by the intravenous route (Rompun, Bayer, Mannhein, Germany) and then immediately euthanized by an intravenous overdose of embutramide and mebezonium iodide (T61, Intervet, Salamanca, Spain).

### Clinical monitoring

Pregnant sheep were examined twice a day by a veterinarian. Rectal temperatures were measured daily for 14 days after infection and then on days 20 and 28 pi. Rectal temperatures greater than 40 °C were considered fever [29]. Foetal viability was assessed daily by ultrasound scanning (monitoring the foetal heartbeat and movements) until day 14 pi and every 4 days thereafter.

### In vivo collection of samples

Blood samples were collected from groups euthanized on day 28 pi (G3 and G5) by jugular venipuncture every day between day 0 and 10 pi and on days 13, 20 and 28 pi in 5 mL vacutainer tubes without anticoagulant and with heparin as an anticoagulant (Becton Dickinson and Company, Plymouth, UK). Serum was obtained from tubes without anticoagulant after centrifugation at 405 × *g* for 10 min at 4 °C and stored at −80 °C until use.

### Post-mortem collection of samples

After euthanasia, the intestine was removed from the abdominal cavity, and the mesenteric lymph nodes were visually inspected. The jejunal mesenteric lymph nodes from the proximal, medial, distal and terminal areas (draining the jejunum and the proximal ileum) and ileocolic lymph node (draining the terminal ileum) [30, 31] were collected for histopathological and molecular studies. In addition, samples from (i) the duodenum (50 cm forwards of the pylorus); (ii) the proximal, medial and distal areas of the jejunum, with and without Peyer’s patches (PPs); (iii) the ileum (70 cm backwards from the ileocecal valve); and (iv) the ileocecal valve were collected. In these areas, the small intestine was sectioned longitudinally to assess macroscopic lesions, and then, 1–2 cm samples were taken, washed twice in PBS, and stored for histopathological and molecular studies.

From the placenta, 12 placentomes (4 each in the cranial, intermediate and distal regions of the uterine horns) were sectioned longitudinally and assessed for macroscopic lesions, and samples were taken for histopathological and molecular studies. In aborted sheep from G3, samples were collected from cotyledons for molecular studies but not for histopathological studies because of autolysis. In the foetuses, the liver and lungs were collected for molecular studies, and the brain was collected for histopathological studies. All samples collected for molecular studies were stored at −80 °C until use. For histopathological examinations, tissue samples were fixed for 5 days in 10% buffered formalin.

### Histopathology

Tissue samples fixed in 10% buffered formalin were trimmed to obtain one section per organ/sample, except for the foetal brain, for which four different sections were obtained (frontal lobe, corpus callosum, midbrain and cerebellum) as previously described [32]. The samples were processed conventionally for histological studies and stained with haematoxylin and eosin. Additionally, in order to increase the sensitivity of leukomalacia detection, one section from each paraffin block of foetal brains was immunolabelled for beta amyloid precursor protein (βAPP) as described previously [32] using a primary monoclonal antibody against βAPP (1:35 000, clone 22C11, Millipore) and counterstained with Mayer’s haematoxylin. The microscopic lesions were examined under an optical microscope (Nikon^®^ Eclipse E600 microscope) coupled with a Nikon^®^ DS-Fi1 digital camera (Nikon, Tokyo, Japan). Lesions were recorded by two veterinary pathologists.

Histopathological findings for the small intestine and mesenteric lymph nodes were scored as follows: “ + ” was assigned to histological changes without pathological significance, such as congestion. A score of “ +  + ” was given to nonspecific lesions, including mild nonsuppurative inflammatory infiltrates of the epithelium and lamina propria, as well as intraepithelial follicles or reactive follicles in the PPs for the small intestine and reactions in the cortical and/or medullary regions for mesenteric lymph nodes. Lesions consistent with *T. gondii* infection, such as granulomatous enteritis in the small intestine and granulomas in mesenteric lymph nodes, were classified as “ +  +  + ” due to their greater pathological relevance.

Histological findings in the placentomes were categorised into three grades on the basis of their pathological significance. Mild histological changes without clear pathological relevance were classified as “ + ” and included findings such as calcifications or the accumulation of cellular debris between villi. Nonspecific histological changes were designated as “ +  + ” and included features such as vascular congestion, endothelial activation, intravascular coagulation, or pyknosis of the maternal epithelium. Vascular lesions with greater pathological significance were classified as “ +  +  + ”, including lesions of the vascular walls, haemorrhages and thrombi. Histopathological changes in the foetal brain were classified as “ + ” for congestion and/or haemorrhage, “ +  + ” for leukomalacia and “ +  +  + ” gliosis.

### DNA extraction and PCR for parasite DNA detection and quantification

Genomic DNA was extracted using the commercial Maxwell^®^ 16 Mouse Tail DNA Purification Kit (Promega, WI, USA) following the manufacturer's instructions. The samples processed included: i) three samples (50–100 mg each) from each of the different areas of the small intestine, mesenteric lymph nodes, and liver and lungs of the foetuses; ii) one sample from three placentomes (1 each in the cranial, intermediate and distal regions of the uterine horns) or three cotyledons from aborted sheep in G3; and iii) one sample from white blood cells (see the “Purification of white blood cells” section). The DNA concentration of each sample was determined using a Synergy® H1 multimode microplate reader (Biotek, Winooski, USA) and Gen5 version 2.09.1 software (Biotek, Winooski, USA) and adjusted to 100 ng/µL. *Toxoplasma gondii* DNA detection was carried out by nested PCR of the ITS-1 region [33]. *T. gondii* DNA in positive samples from the small intestine, mesenteric lymph nodes and white blood cells, identified by nested PCR, was quantified using qPCR as previously described [8]. The parasite burden in the tissue samples was expressed as the parasite number/mg ovine tissue.

### Purification of white blood cells

The white blood cells were purified as previously described [34], with modifications. Briefly, 5 mL of blood collected in vacutainer tubes containing heparin was centrifuged at 650 × *g* for 30 min at room temperature without acceleration or deceleration, and the buffy coat, containing white blood cells, was collected. To lyse the remaining red blood cells, the buffy coat was washed three times in Milli-Q water containing 0.03% sodium bicarbonate, each of which was followed by centrifugation at 12 000 × *g* for 1 min at 4 °C. After resuspension in PBS and centrifugation at 12 000 × *g* for 1 min at 4 °C, the pellet containing white blood cells was stored at –80 °C until use.

### Anti-*T. gondii* IgG levels in the dam’s sera

*Toxoplasma gondii*-specific IgG levels were determined by a previously validated in-house TgSALUVET ELISA 2.0 [35]. Briefly, 96-well microtiter plates (Maxisorp^®^, Thermo Scientific, MA, USA) were coated overnight at 4 °C with 100 μL of carbonate buffer containing 10^5^ lyophilized tachyzoites per well. The plates were blocked, and the serum samples were diluted 1:100 with 5% powdered skim milk in PBS containing 0.05% Tween 20 (PBS-T). Subsequently, a monoclonal anti-goat/sheep IgG antibody conjugated with peroxidase (Sigma, MA, USA) diluted 1:10 000 in PBS-T was added, followed by the use of ABTS (Roche, Basilea, Switzerland) as a substrate. The reaction was stopped with 0.3 M oxalic acid, and the optical density (OD) was read at 405 nm. For each plate, values of the OD were converted into a relative index percent (RIPC) using the following formula: RIPC = (OD sample—OD negative control)/(OD positive control–OD negative control) × 100. A RIPC value ≥ 32.21 indicates a positive result.

### Statistical analysis

Rectal temperatures and *T. gondii*-specific IgG levels in the dams were compared between groups using two-way repeated-measures ANOVA. Differences in parasite DNA detection in the small intestine and mesenteric lymph nodes between different zones or between the different infection groups were evaluated using Fisher’s exact F test. The correlation between the categories of histopathological lesions and the parasite DNA detection rates in the small intestine and mesenteric lymph nodes was evaluated separately for each infected group by calculating Spearman’s correlation coefficient. Differences in parasite burdens were analysed via the nonparametric Kruskal‒Wallis test followed by Dunn’s test and the Mann‒Whitney test for pairwise comparisons. Statistical significance for all analyses was established at *P* < 0.05. Differences in parasite DNA detection rates and parasite burdens with *P* values ≥ 0.05 and ≤ 0.10 were considered to have a tendency towards statistical significance. All the statistical analyses were performed using GraphPad Prism 8.01 software (San Diego, CA, USA).

## Results

### Rectal temperatures

**Figure 2:**
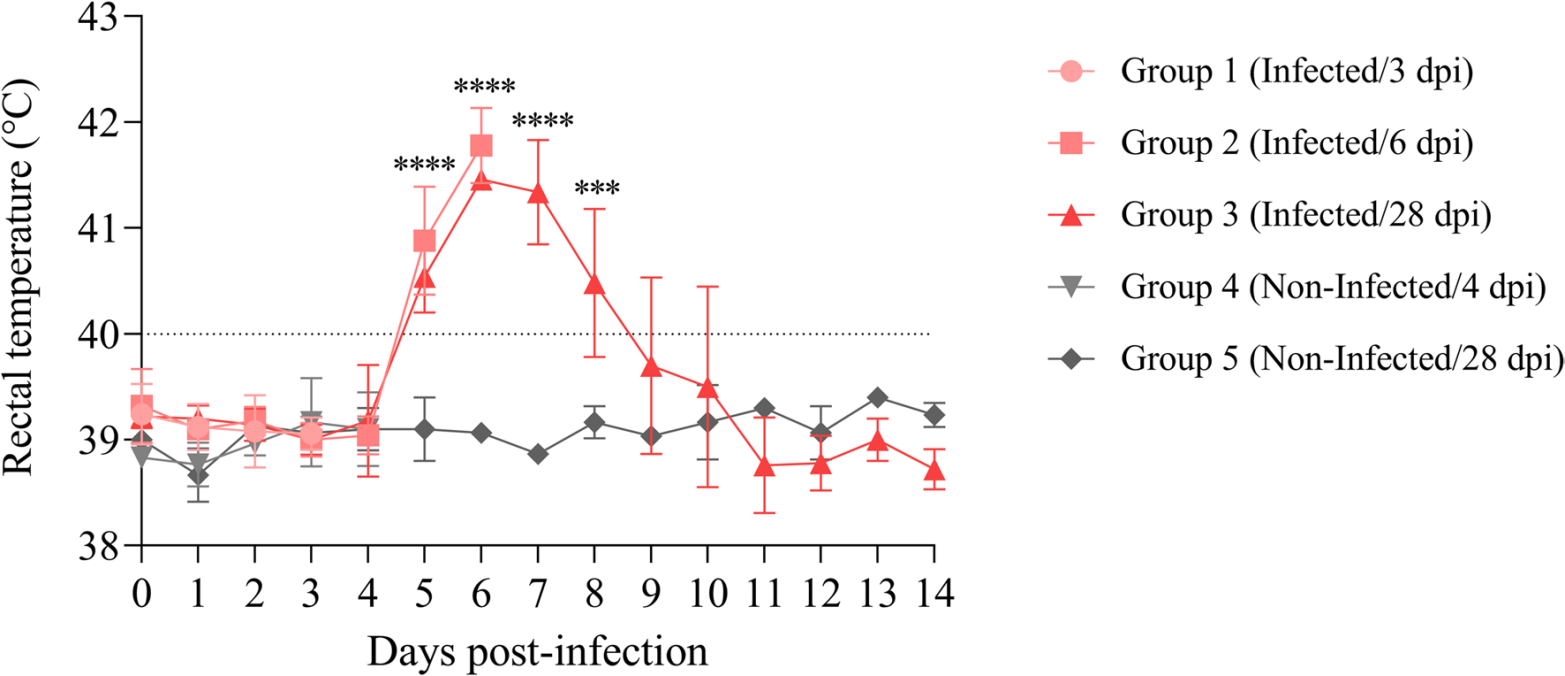
**Rectal temperatures of** ***T. gondii*-infected and uninfected sheep. Each point represents the mean ± S.D. for each group.** Rectal temperatures were analysed using two-way repeated-measures ANOVA. For significant differences, (***) indicates *P* < 0.001 and (****) indicates *P* < 0.0001.

Rectal temperatures of infected sheep euthanised on day 3 pi (G1) were within the physiological range and did not differ significantly from those of uninfected sheep. In the infected groups euthanised on days 6 and 28 pi (G2 and G3, respectively), all animals developed fever and statistically significant increases in rectal temperature were observed on days 5 (*P* < 0.0001) and 6 pi (*P* < 0.0001) compared with those in uninfected sheep (G5) (Figure 2). Additionally, sheep euthanised on day 28 pi (G3) maintained higher rectal temperatures than uninfected sheep on days 7 (*P* < 0.0001) and 8 pi (*P* < 0.001) (Figure 2).

### Foetal survival

All the foetuses from infected sheep euthanised on days 3 and 6 pi (G1 and G2) were alive (heartbeat and movements by ultrasound scanning) at the time of euthanasia. In G3 (infected sheep), foetal death was detected on day 8 pi in 4 out of 5 sheep (80%), which aborted on days 10 and 11 pi (early abortions), while foetuses from the remaining sheep were alive at the time of euthanasia (28 dpi). All foetuses from uninfected sheep (G4 and G5) were alive prior to the euthanasia of the dams.

### *Toxoplasma gondii* DNA in maternal white blood cells

Only 1 out of 5 animals from G3 (infected/euthanized on day 28 pi) was PCR positive by nested PCR (but negative by qPCR) on day 10 pi. The remaining infected sheep and the uninfected sheep were negative.

### Humoral immune response

**Figure 3:**
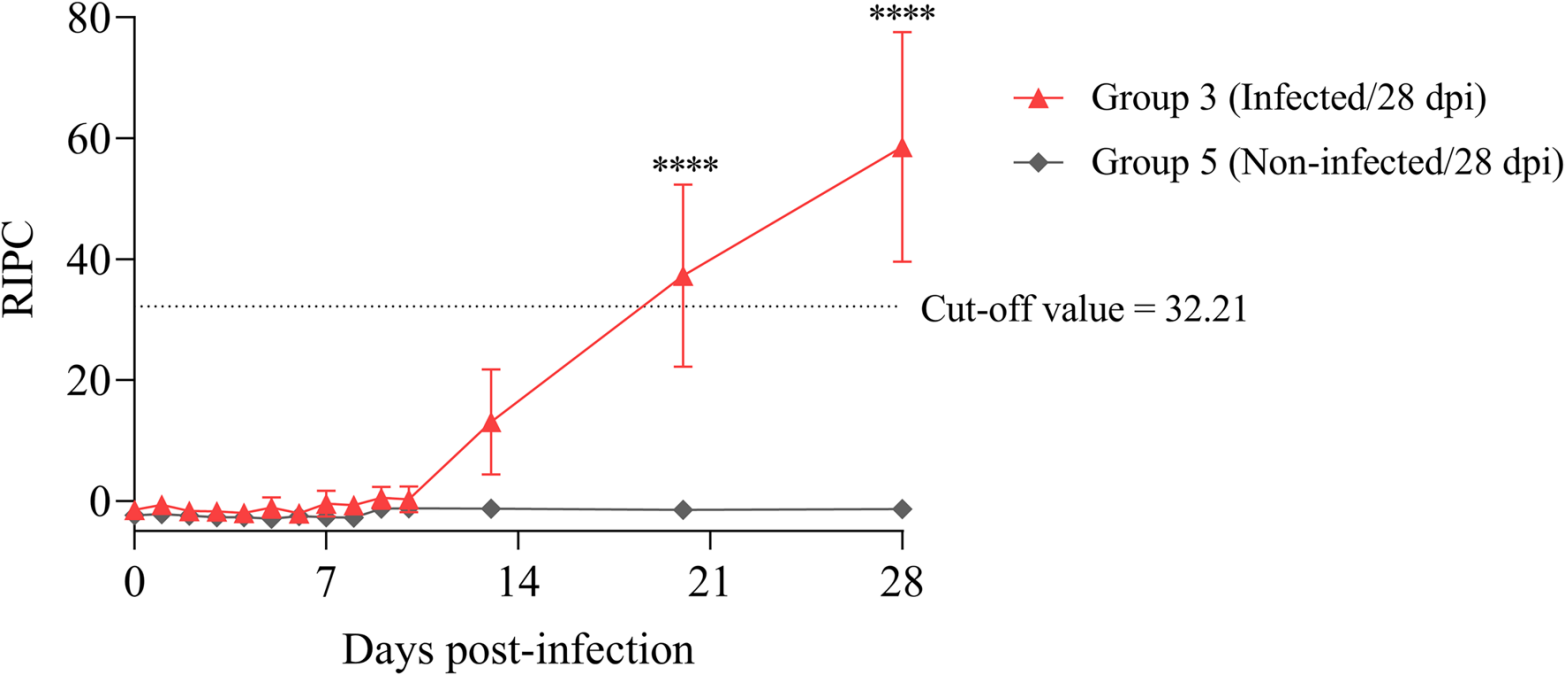
**Anti-*T. gondii* IgG levels in the dam’s sera. IgG levels are expressed as the relative index percent (RIPC).** Each point represents the mean ± S.D. at the different sampling times for each group. The horizontal dashed line in B indicates the cut-off (RIPC ≥ 32.21) for the validated TgSALUVET ELISA 2.0 [35]. *Toxoplasma gondii*-specific IgG levels in the dams were analysed using two-way repeated-measures ANOVA. For significant differences between the infected and uninfected groups, (****) indicates *P* < 0.0001.

Anti-*T. gondii* IgG levels were above the cut-off value for 4 out of 5 infected animals in G3 on day 20 pi and for all the animals in this group on day 28 pi (Figure 3). Uninfected sheep from G5 remained at basal anti-*T. gondii* IgG levels throughout the study.

### Lesions, and parasite DNA detection and loads in the small intestine

Macroscopic observations of the small intestine of infected animals did not reveal significant lesions, with only 2 out of 5 animals euthanized on day 6 pi exhibiting petechiae in the proximal jejunum or congestion of the distal jejunum. Macroscopic lesions were not observed in the small intestine of uninfected animals. Histological examination of the intestinal samples revealed mild nonsuppurative inflammatory infiltrates within the epithelium and lamina propria (+ +) in at least 50% of the infected animals in the following intestinal regions: proximal jejunum (PP) and medial jejunum in infected animals euthanized on day 3 pi (G1), proximal jejunum (PP) and medial and distal jejunum (wall and PP in both regions) in infected animals euthanized on day 6 pi (G2) and proximal jejunum (PP) and medial jejunum in infected animals euthanized on day 28 pi (G3) (Table 1; Additional file 1).

**Table 1:** **Overview of histopathological lesions and parasite DNA detection in small intestine sections.**

			Histological score	Duodenum	Jejunum						Ileum	Ileocecal valve
					Proximal		Medial		Distal			
					Wall	Wall + PP	Wall	Wall + PP	Wall	Wall + PP		
Infected groups	Group 1 (3 dpi)	Histopathological lesions	+	0/5	0/5	0/5	0/5	0/4	0/5	2/5	0/5	1/5
			+ +	0/5	1/5	3/5	3/5	0/4	0/5	0/5	0/5	0/5
			+ + +	0/5	0/5	0/5	0/5	0/4	0/5	0/5	2/5	2/5
		Parasite DNA detection (PCR)		0/5	**1/5 (1)**	**1/5 (1)**	**1/5 (1)**	**1/5 (1)**	0/5	0/5	0/5	0/5
	Group 2 (6 dpi)	Histopathological lesions	+	0/5	0/5	0/3	0/5	0/4	1/4	1/5	1/5	0/4
			+ +	2/5	1/5	2/3	3/5	3/4	2/4	3/5	0/5	0/4
			+ + +	0/5	0/5	0/3	0/5	0/4	0/4	0/5	0/5	1/4
		Parasite DNA detection (PCR)		**1/5 (1)**	0/5	0/5	0/5	**3/5 (1, 2, 3)**	**2/5 (1, 2)**	0/5	**1/5 (1)**	0/5
	Group 3 (28 dpi)	Histopathological lesions	+	0/5	0/5	0/4	2/5	0/5	0/5	0/5	0/5	0/4
			+ +	1/5	2/5	2/4	3/5	2/5	0/5	0/5	0/5	0/4
			+ + +	0/5	0/5	0/4	0/5	0/5	0/5	0/5	0/5	0/4
		Parasite DNA detection (PCR)		0/5	**1/5 (1)**	**1/5 (1)**	**3/5 (1)**	**2/5 (2, 3)**	**1/5 (1)**	**2/5 (2, 3)**	**1/5 (2)**	**1/5 (1)**
Noninfected groups	Group 4 (4 dpi)	Histopathological lesions	+	0/3	0/3	0/3	0/3	0/3	2/3	0/3	0/3	0/3
			+ +	0/3	0/3	1/3	0/3	0/3	0/3	0/3	1/3	0/3
			+ + +	0/3	0/3	0/3	0/3	0/3	0/3	0/3	0/3	0/3
		Parasite DNA detection (PCR)		0/3	0/3	0/3	0/3	0/3	0/3	0/3	0/3	0/3
	Group 5 (28 dpi)	Histopathological lesions	+	0/3	0/3	0/2	0/3	0/2	0/3	0/3	0/3	0/3
			+ +	1/3	1/3	1/2	2/3	1/2	0/3	0/3	0/3	0/3
			+ + +	0/3	0/3	0/2	0/3	0/2	0/3	1/3	0/3	0/3
		Parasite DNA detection (PCR)		0/3	0/3	0/3	0/3	0/3	0/3	0/3	0/3	0/3

*dpi* days post-infection when the animals were euthanized. *PP* Peyer’s patch. The numbers represent the number of animals with histopathological lesions or PCR-positive samples/total number of animals. Histopathological score: “ + ” histological changes without pathological significance, such as congestion; “ +  + ” nonspecific lesions, including mild nonsuppurative inflammatory infiltrates of the epithelium and lamina propria as well as intraepithelial follicles or reactive follicles in the PP; “ +  +  + ” lesions consistent with *T. gondii* infection, such as granulomatous enteritis. Tissues in which parasite DNA was detected are shown in bold, with the number of positive replicates among the three replicates analysed per tissue and animal in brackets

On day 3 pi, *T. gondii* DNA was sporadically detected (4/134, 3%) in proximal and medial jejunum samples (both wall and PP) from 2 out of 5 animals (Table 1). The parasite burdens were low, and only one positive sample from the PP in the medial jejunum could be quantified (3.45 tachyzoites/mg) (Additional file 1). On day 6 pi, *T. gondii* DNA positivity was also scarcely detected (11/135, 8.1%), but 4 out of 5 animals were positive in some samples. In the PP from the medial jejunum, parasite DNA was detected in several replicates in 3 out of 5 animals. This section presented significantly higher parasite DNA detection rates (*P* < 0.05) than the other small intestinal sections, with the exception of the distal jejunum section, where the parasite DNA was detected in 2 out of 5 animals (Table 1). In terms of parasite burden, 4 out of the 5 positive samples identified by qPCR were PPs in the medial jejunum and presented a parasite burden of 44.9 ± 54.3 tachyzoites/mg (Additional file 1). On day 28 pi, parasite DNA was also detected in 4 out of 5 animals (19/135 positive samples, 14.1%) (Table 1). Concerning parasite burdens, 5 samples were quantified: 3 from the PP of the medial jejunum (3.35 ± 0,59 tachyzoites/mg), 1 from the PP of the distal jejunum (36 tachyzoites/mg) and 1 from the ileocecal valve (2.4 tachyzoites/mg) (Additional file 1).

We next compared the infection dynamics on different days pi in the same section. In the PP from the medial jejunum, higher parasite DNA detection rates were found on day 6 pi than on day 3 pi, although this difference was not statistically significant (*P* = 0.08). Moreover, no significant differences in parasite DNA detection rates in PPs from the medial jejunum were found between days 6 and 28 pi. The parasite burden from the medial jejunal PP section tended to be significantly greater on day 6 pi than on day 3 pi (*P* = 0.10). In the distal jejunal PP section, higher parasite DNA detection rates were detected on day 28 pi. than on days 3 and 6 pi (*P* < 0.05). There was no correlation between categories of histopathological lesions and parasite DNA detection rates (Spearman’s r = 0.022 for G1; 0.123 for G2; and 0.192 for G3).

### Lesions, and parasite DNA detection and loads in the mesenteric lymph nodes

**Figure 4:**
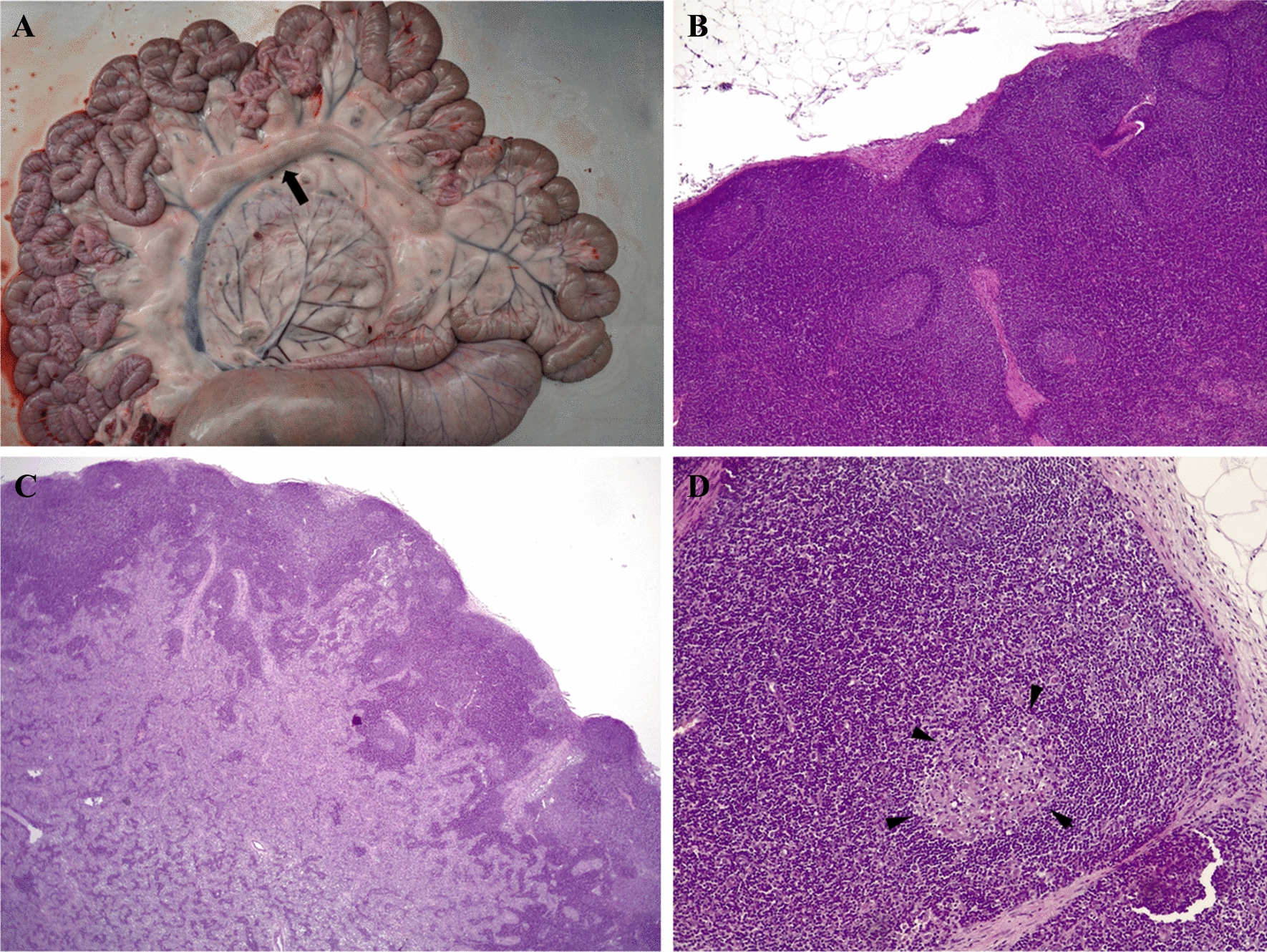
**Macroscopic and microscopic findings in mesenteric lymph nodes.** **A** Adenomegaly of mesenteric lymph nodes (arrow) of sheep 2.1 euthanized on day 6 pi (**B**) Secondary lymphoid follicles at the cortex of a jejunal mesenteric lymph node in sheep 1.3 (infected/euthanized on day 3 pi). H/E, magnification: 40x. **C** Expansion of the medullary sinuses due to increased accumulation of macrophages (sinus histiocytosis) in a jejunal mesenteric lymph node in sheep 2.1 (infected/euthanized on day 6 pi). H/E, magnification: 12x. **D** Granuloma at the cortex of a jejunal mesenteric lymph node (arrowheads) in sheep 3.3 (infected/euthanized on day 28 pi). H/E, magnification: 100x.

Concerning macroscopic lesions, on day 3 pi, 1 out of 5 animals exhibited adenomegaly in the medial and distal jejunal mesenteric lymph nodes. On day 6 pi, all the animals presented adenomegaly in the jejunal lymph nodes (Figure 4A). Furthermore, adenomegaly was found in the ileocolic lymph nodes of 1/5 of the animals. On day 28 pi, no macroscopic lesions were found in the mesenteric lymph nodes. With respect to the presence of histological changes, some jejunal and ileocolic lymph nodes from G1 (infected/euthanized on day 3 pi) and most of the jejunal and ileocolic lymph nodes from G2 (infected/euthanized on day 6 pi) presented numerous secondary lymphoid follicles characterised by prominent germinal centres surrounded by a mantle zone within the cortical region (secondary follicular hyperplasia) (Figure 4B; Table 2; Additional file 2). Additionally, 2 out of 5 sheep from G1 (euthanized on day 3 pi) and 3 out of 5 sheep from G2 (euthanized on day 6 pi) exhibited marked expansion of the medullary sinuses due to an increased accumulation of macrophages (sinus histiocytosis) (Figure 4C). Furthermore, 3 out of 5 sheep from G3 (euthanized on day 28 pi) presented small, isolated granulomas within the cortical region (Figure 4D; Table 2; Additional file 2). These granulomas were composed of aggregates of epithelioid macrophages.

**Table 2:** **Overview of histopathological lesions and parasite DNA detection in lymph nodes draining the small intestine**

				Jejunal mesenteric lymph nodes				Ileocolic lymph nodes
			Histological score	Proximal	Medial	Distal	Terminal	
Infected groups	Group 1 (3 dpi)	Histopathological lesions	+	0/5	0/5	1/5	1/5	0/5
			+ +	2/5	1/5	2/5	2/5	5/5
			+ + +	0/5	0/5	0/5	0/5	0/5
		Parasite DNA detection (PCR)		1/5 (1)	3/5 (1,2)	1/5 (2)	0/5	1/5 (1)
	Group 2 (6 dpi)	Histopathological lesions	+	1/5	2/5	1/5	2/5	0/5
			+ +	2/5	3/5	4/5	1/5	3/5
			+ + +	0/5	0/5	0/5	0/5	0/5
		Parasite DNA detection (PCR)		3/5 (1, 3)	5/5 (1, 2, 3)	5/5 (2, 3)	4/5 (2, 3)	0/5
	Group 3 (28 dpi)	Histopathological lesions	+	0/5	0/5	0/5	0/5	0/5
			+ +	1/5	1/5	0/5	1/5	4/5
			+ + +	1/5	2/5	1/5	0/5	0/5
		Parasite DNA detection (PCR)		2/5 (2)	4/5 (1,3)	5/5 (1, 2, 3)	3/5 (2, 3)	3/5 (1, 2)
Noninfected groups	Group 4 (4 dpi)	Histopathological lesions	+	0/3	0/3	0/3	0/3	0/3
			+ +	0/3	0/3	0/3	0/3	0/3
			+ + +	0/3	0/3	0/3	0/3	0/3
		Parasite DNA detection (PCR)		0/3	0/3	0/3	0/3	0/3
	Group 5 (28 dpi)	Histopathological lesions	+	0/3	0/3	0/3	0/3	0/3
			+ +	1/3	1/3	1/3	1/3	1/3
			+ + +	0/3	0/3	0/3	0/3	0/3
		Parasite DNA detection (PCR)		0/3	0/3	0/3	0/3	0/3

*dpi* days post-infection when the animals were euthanized. Animals with histopathological lesions or PCR-positive samples/total number of animals. Histopathological score: “ + ” histological changes without pathological significance, such as congestion; “ +  + ” nonspecific lesions, such as reactions in the cortical and/or medullary regions; “ +  +  + ” lesions compatible with *T. gondii* infection, such as granulomas. For parasite DNA detection, the number of positive replicates among the three replicates analysed per tissue and animal are shown in brackets.

**Figure 5:**
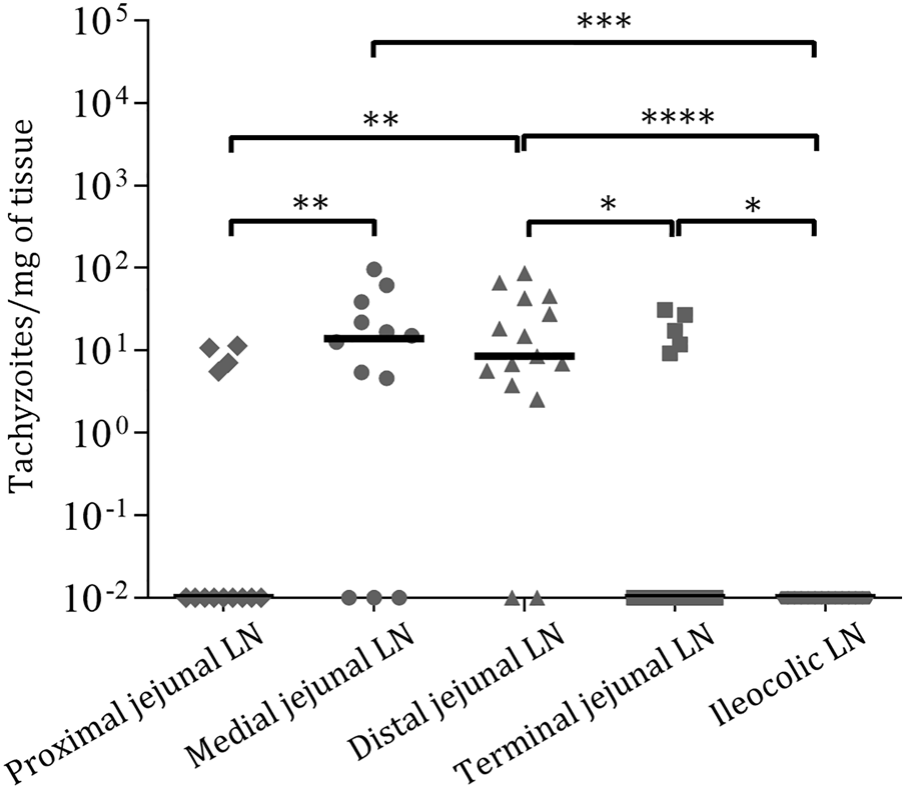
**Dot-plot graphs of the *T. gondii* burden in mesenteric lymph nodes from sheep in G2 (infected/euthanized on day 6 pi).** Each dot represents an individual value of the estimated parasite burden (number of parasites per milligram of host tissue), and the medians are represented as horizontal lines. Considering that the *T. gondii* detection limit by real-time PCR is 0.1 parasites, negative samples (0 parasites) were represented on the log scale as < 0.1 (i.e., 10.^−2^). Parasite burdens were analysed via the nonparametric Kruskal‒Wallis test followed by Dunn’s test for comparisons between different lymph nodes, as well as the Mann‒Whitney test for pairwise comparisons. For significant differences between infected groups in each tissue, (*) indicates *P* < 0.05, (**) indicates *P* < 0.01, (***) indicates *P* < 0.001 and (****) indicates *P* < 0.0001.

On day 3 pi, 8 positive samples out of 73 (11%) were detected via nested PCR (Table 2). The parasite burdens were too low to be quantified (Additional file 2). On day 6 pi, parasite DNA detection was greater, with 61.2% of the samples being positive. Medial and distal mesenteric lymph nodes presented higher parasite DNA detection rates than the proximal jejunal mesenteric lymph nodes (*P* < 0.01). None of the samples from the ileocolic lymph nodes were positive for *T. gondii* DNA. With respect to parasite burdens, the medial and distal jejunal mesenteric lymph nodes (30.13 ± 30.32 tachyzoites/mg and 25.75 ± 26.72 tachyzoites/mg, respectively) presented significantly greater parasite burdens than the proximal jejunal mesenteric lymph nodes (8.64 ± 2.84 tachyzoites/mg), terminal jejunal mesenteric lymph nodes (19.16 ± 9.41 tachyzoites/mg) and ileocolic lymph nodes (lack of positive samples) (Figure 5; Additional file 2). On day 28 pi, 37 out of 70 (52.9%) positive samples were detected via nested PCR. Compared with proximal jejunal mesenteric lymph nodes, medial and distal jejunal mesenteric lymph nodes presented higher parasite DNA detection rates (*P* = 0.06 and *P* < 0.05, respectively). Only 7 samples were quantified, 4 of which were from the medial jejunal mesenteric lymph nodes, with parasite burdens of 8.48 ± 7.61 tachyzoites/mg. There were no statistically significant differences in parasite burdens between different lymph nodes (Additional file 2).

When we compared the dynamics of infection on different days pi in the mesenteric lymph nodes draining the same small intestinal area, all the lymph nodes, except the ileocolic lymph nodes, presented significantly higher parasite DNA detection rates on days 6 and 28 than on day 3 pi. For the ileocolic lymph nodes, the parasite DNA detection rates were higher on day 28 pi than on days 3 and 6 pi (*P* < 0.05). In terms of parasite burden, the medial and distal jejunal mesenteric lymph nodes presented greater values on day 6 than on days 3 and 28 pi (*P* < 0.05) (Additional file 2). There was no correlation between categories of histopathological lesions and parasite DNA detection rates in G1 and G3 (Spearman’s r = −0.068 and 0.071, respectively), whereas in G2, a very weak correlation was observed (Spearman’s r = 0.357).

### Lesions and parasite DNA detection in the placenta

**Figure 6:**
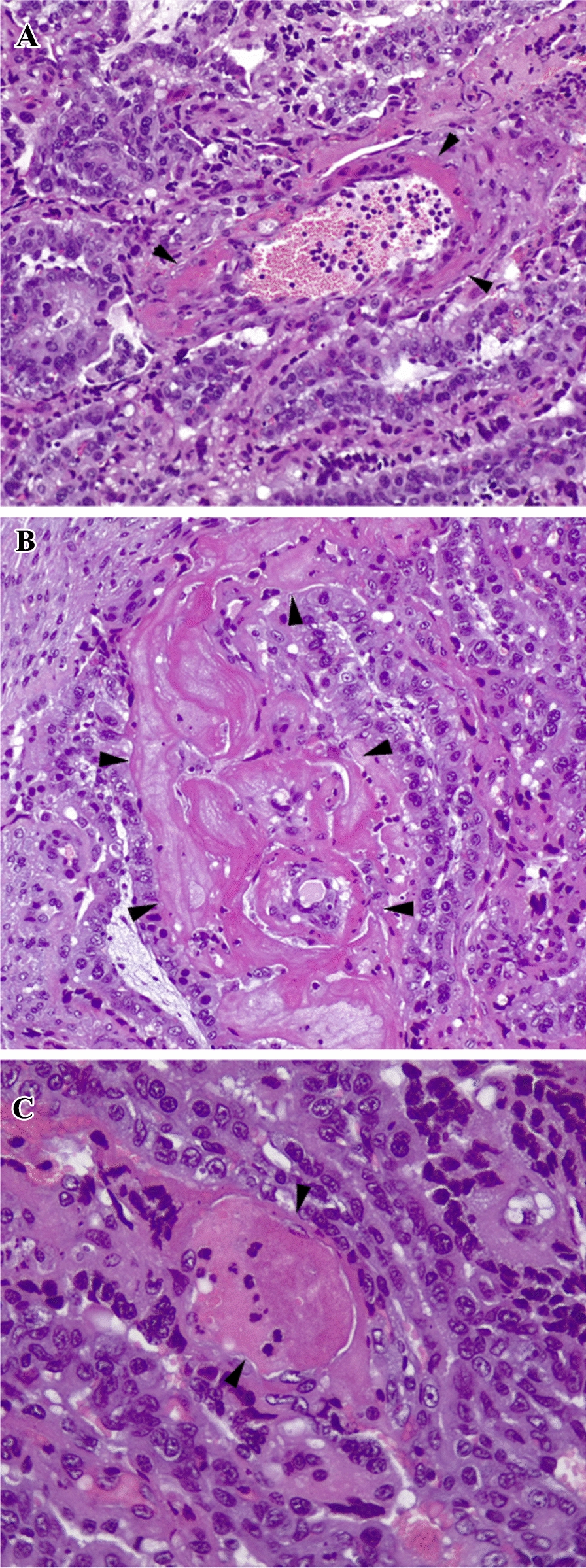
**Histological lesions in the placentomes.** **A** Hyaline degeneration of a vessel wall in a maternal villus at the placenta denoted by a deposit of homogeneous, eosinophilic material in the wall (arrowheads). Sheep 1.2 (infected/euthanized on day 3 pi). H/E. Magnification: 200x. **B** Marked extravasation of eosinophilic fibrin, which had undergone polymerisation, forming dense fibrillar deposits within the surrounding stroma (arrowheads). Sheep 2.2 (infected/euthanized on day 6 pi). H/E. Magnification: 200x. **C** Caruncular vessel within the interdigitated area of the placentome completely occluded by a thrombus (arrowheads). Note the hyaline degeneration of the vascular wall at the affected vessel. Sheep 2.2 (infected/euthanized on day 6 pi). H/E. Magnification: 400x.

In infected sheep euthanized on day 3 pi (G1) and 6 pi (G2) and in uninfected sheep euthanized on day 4 pi (G4) and 28 pi (G5), macroscopic lesions compatible with placental infarcts were not observed. Macroscopic observations could not be carried out in placental tissues from sheep suffering early abortion (G3) due to the autolysis of cotyledons. In infected sheep carrying live foetuses on day 28 pi, macroscopic lesions were not observed in the placentomes. Histological lesions of pathological significance were found mainly in the placentomes of sheep euthanized on day 6 pi (G2) (Table 3; Additional file 3). Microscopic lesions categorized as “ +  + ”, such as congestion, endothelial activation, coagulation and maternal epithelium pyknosis, were observed in 6.3% (3/48) of the placentomes from G1 (2/5, 40% of the infected sheep euthanized on day 3 pi with at least one placentome showing these lesions), in 18.6% (11/59) of the placentomes from G2 (4/5, 80% of the infected sheep euthanized on day 6 pi with at least one placentome showing these lesions), and in 8.3% (6/72) of the placentomes from uninfected sheep (G4 and G5), with 2/6 sheep (33%) showing at least one placentome with these lesions. In the affected vessels, the endothelial cells appeared attenuated, and the surrounding villous stroma displayed mild oedema (Figure 6A). Occasionally, there was a marked extravasation of eosinophilic fibrin, which formed dense fibrillar deposits within the surrounding stroma, leading to marked narrowing of the vessel lumen (Figure 6B). A varying degree of occlusion by thrombi in the vessels (categorized as “ +  +  + ”) (Figure 6C) was observed in 2 out of 5 sheep (40%) from G2 (infected/euthanized on day 6 pi), as exhibited by 3/12 placentomes of sheep G2.2 and 1/12 placentomes of sheep G2.3 (Table 3; Additional file 3). In the only non-aborted sheep from G3 (infected/euthanized on day 28 pi), microscopic lesions in the placentomes included mainly inflammatory foci (5/12 placentomes); however, some placentomes (2/12) also presented congestion, endothelium activation and coagulation (Additional file 3).

**Table 3:** **Histological lesions in placentomes**

		Histological score	Total
Infected groups	Group 1 (3 dpi)	+	10/48 (20.8%)
		+ +	3/48 (6.3%)
		+ + +	0/48 (0%)
	Group 2 (6 dpi)	+	12/59 (20.3%)
		+ +	11/59 (18.6%)
		+ + +	4/48 (8.3%)
Noninfected groups	Group 4 (4 dpi)	+	18/36 (50%)
		+ +	5/36 (13%)
		+ + +	0/36 (0%)
	Group 5 (28 dpi)	+	10/36 (27.8%)
		+ +	1/36 (2.8%)
		+ + +	0/36 (0%)

dpi: days post-infection when the animals were euthanized. Placentomes with histopathological lesions/total number of placentomes evaluated, with percentages between brackets. Histopathological score: “ + ” mild histological changes without clear pathological relevance, including findings such as calcifications or the accumulation of cellular debris between villi; “ +  + ” nonspecific histological changes, including features such as vascular congestion, endothelial activation, intravascular coagulation, or pyknosis of the maternal epithelium; “ +  +  + ” vascular lesions with greater pathological significance, including lesions of the vascular walls, haemorrhages and thrombi.

Parasite DNA was not detected in placentomes from infected sheep euthanised on days 3 and 6 pi (G1 and G2) or in cotyledons from infected sheep in G3 suffering foetal death on day 8 pi. In contrast, all placentomes from the sheep in G3 carrying live foetuses at 28 days pi were PCR positive.

### Lesions and parasite DNA detection in foetal tissues

**Figure 7:**
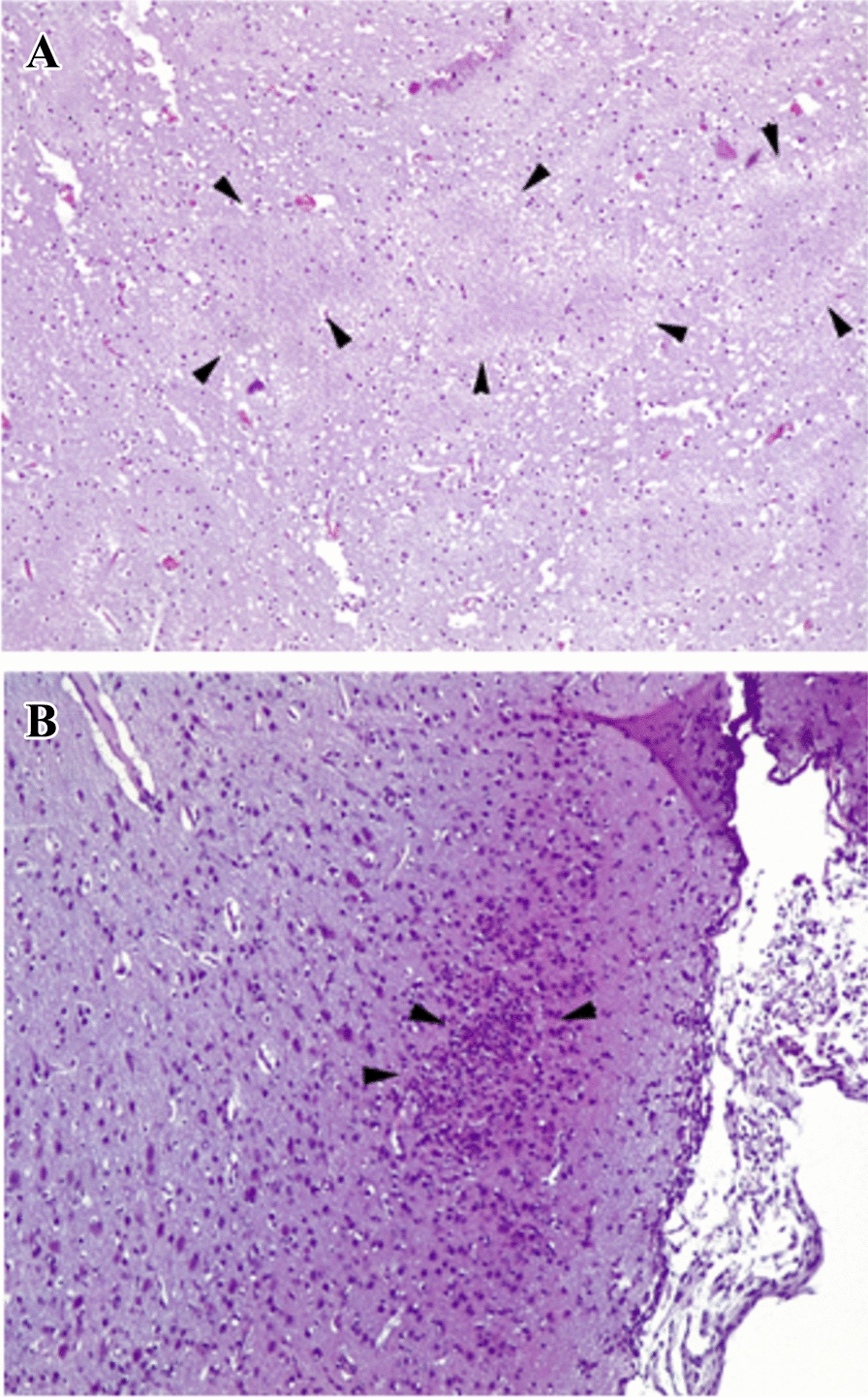
**Histological lesions in the foetal brains.** **A** Areas of leukomalacia (arrowheads) in the white matter of a foetal brain from an early abortion in G3. **B** Focus of glial cells (arrowheads) in the brain of a live foetus on day 28 pi in G3.

Lesions of leukomalacia were not observed in foetuses from sheep in G1 and G2 (infected and euthanized on days 3 and 6, respectively). In foetuses that experienced early abortion on day 8 pi (G3), leukomalacia was observed on some slides from 2 out of 14 foetuses (14.3%) (Figure 7A), but most of the samples were autolytic, preventing a proper histological evaluation. On the other hand, 2 out of 3 of the foetuses that remained alive until day 28 pi in G3 presented microscopic lesions consisting of glial foci in most of the studied sections (Figure 7B) (Table 4). βAPP labelling of foetal brains revealed two different patterns: (i) staining of well demarcated areas of variable size within the white matter and (ii) diffuse areas where the cytoplasm of different cell populations (mainly neurons, microglia, astrocytes and Purkinje cells in the cerebellum) were labelled (Additional file 4; Additional file 5). The first type of labelling (well demarcated areas of white matter) was observed only in foetuses (10/11, 90.1%) that died on day 8 pi (G3). The second type of staining (cytoplasmic labelling) was observed in a few slides from 4/10 (40%) G1 foetuses (infected and euthanized on day 3 pi) and 5/8 (62.5%) G2 foetuses (infected and euthanized on day 6 pi), as well as in all 3 foetuses that remained alive at 28 dpi from the only sheep that did not abort in G3. Finally, one slide each of 4/11 (36.4%) foetuses from G3 that was aborted on day 8 pi presented both types of labelling, with an evident cytoplasmic labelling of cells morphologically compatible with astrocytes. βAPP immunolabelling was not observed in any of the slides from foetuses of the uninfected sheep (G4 and G5).

**Table 4:** **Histological lesions in foetal brains.**

		Sheep ref	Foetus 1				Foetus 2				Foetus 3			
			Slide A	Slide B	Slide C	Slide A	Slide B	Slide C	Slide A	Slide B	Slide C	Slide A	Slide B	Slide C
Infected groups	Group 1 (3 dpi)	1.1												
		1.2							+					
		1.3												
		1.4							+					
		1.5			+									
	Group 2 (6 dpi)	2.1		+										
		2.2					+				+			
		2.3												
		2.4												
		2.5												
	Group 3 (28 dpi)	3.1*	A	A	**A + + **		A	A	A					
		3.2*	A +	A +	A		A +	A			A	A +	A	
		3.3*	A +				A +	A +			A			
		3.4	+ + +		+ + +	+ + +		+ + +	+ + +	+ + +				
		3.5*					A	A			**A + + **	**A + + **	A	
Noninfected groups	Group 4 (4 dpi)	4.1					+							
		4.2												
		4.3			+									
	Group 5 (28 dpi)	5.1												
		5.2												
		5.3												

dpi: days post-infection when the animals were euthanized. “*” sheep with foetal death on day 8 post-infection. A: autolytic samples. Slides A, B, C and D belong to frontal lobe, corpus callosum, midbrain and cerebellum, respectively. Histopathological score: “ + ” congestion and haemorrhage; “ +  + ” leukomalacia (in bold); “ +  +  + ” gliosis.

Parasite DNA was not detected in foetuses from infected sheep euthanised on days 3 pi (G1) and 6 pi (G2) or in foetuses from G3 that died on day 8 pi. Parasite DNA was detected in all livers and lungs analysed from the three foetuses in G3 (infected/euthanised on day 28 pi) that were alive at the time of euthanasia.

## Discussion

Limited information is available regarding the early phase of *T. gondii* infection in pregnant sheep and the host‒parasite interactions that occur following oral ingestion of sporulated oocysts. These interactions within the small intestine, mesenteric lymph nodes and placenta could play crucial roles in the pathogenesis of early abortions, which typically occur during the second week post-infection and whose pathogenesis remains largely unknown [18].

In this study, three different euthanasia times were chosen with the aim of understanding the early dynamics of *T. gondii* infection: (i) day 3 pi as the earliest time point, considering that the intestinal transit time in sheep is approximately 24 h [26]; (ii) day 6 pi, coinciding with the fever peak and two days prior to the detection of the first early foetal death events [18]; and (iii) day 28 pi, to monitor the time course of early abortions, parasite DNA detection in white blood cells, and seroconversion in infected sheep. The rectal temperature increases observed in the present study, with fever in infected animals between days 5 and 8 pi, were similar to those reported in previous studies carried out with the same isolate and dose of infection [36, 37]. The increase in rectal temperature does not seem to be related to the pathogenesis of early abortion, as no difference was observed between aborted and nonaborted sheep in previous studies [16]. In terms of foetal viability and seroconversion, in the infected group maintained for this purpose (G3), 80% of the sheep experienced early abortion on day 8 pi and seroconverted on day 21 pi, similar to previous observations in which sheep were also infected at mid-pregnancy with 1000 TgShSp1 oocysts [37].

*T. gondii* tachyzoites spread rapidly from the initial site of infection in the intestine to distant tissues, migrating from the intestinal mucosa through the lymphatic system and bloodstream, and finally reaching organs such as the placenta [23, 24, 38, 39]. Previous studies in mice have provided a comprehensive picture of the interaction of *T. gondii* with the intestinal barrier. Three days after oral infection, tachyzoites infected diverse cells within the lamina propria of the small intestine [40–44]. However, some studies failed to detect the parasite in the small intestine on day 3 pi [45], or the parasite DNA was detected only in the PP but not in the parenchyma [46], but was detected a few days later, on day 5 pi. In the present study, the detection rate of *T. gondii* DNA in the sheep small intestine was low (3%, 8.1% and 14.1% of the small intestine samples on days 3, 6 and 28 pi, respectively), which may be due to the small amount of intestinal tissue processed (1–2 cm from each zone), given that the sheep small intestine is approximately 26 m long [25]. Previous studies in lambs have shown that live tachyzoites [9] or *T. gondii* DNA [4] can be detected in the small intestine as early as day 4 pi, with a relatively high detection rate at one week post-infection. Similar to mice [46], in our study, parasite DNA detection and burden were greater in PPs than in the parenchyma on day 6 pi. Peyer’s patches (PPs) are part of the gastrointestinal associated lymphoid tissue located at the lamina propria of the intestinal mucosa and submucosa and are rich in dendritic cells and macrophages, the first immune cells to recognise parasite infection and initiate the host immune response*. Toxoplasma gondii* replication in the PP could act as foci for systemic dissemination of the parasite from the gut [21, 23]. At later time points, on day 28 pi, similar to previous studies in lambs [4, 9], the parasite DNA were still detected in the small intestine, although the burden was lower than that at the end of the first week pi, and the positive samples were also mainly from the PP, as in previous studies [4]. In this study, we assessed different regions of the small intestine separately, aiming to trace the initial entry site of *T. gondii* in sheep following oral infection with oocysts. Previous studies in sheep have not examined the different regions of the small intestine separately [9] or used tissue cysts with bradyzoites to infect the animals [4]; hence, the results are not comparable. In mice, most studies have identified the ileum as the main site of parasite replication in the small intestine [40, 41, 47, 48]; however, after infection of C57BL/6 mice with 50 Pru cysts, the highest parasite burdens on day 6 pi were present in the proximal jejunum [44], similar to our findings in sheep. Three days after the infection of sheep with oocysts, parasite DNA and lesions were mainly detected in the proximal and medial jejunum, while on day 6 pi, parasites and lesions were also detected in more distal sections of the small intestine, such as the distal jejunum. This detection of parasite DNA at more distal sites as the infection progresses has been previously reported in the sheep small intestine [4] and may be due to the release of parasites into the gut lumen after lysis of intestinal epithelial cells and the viability of parasites in the gut lumen for several days [22, 44]. With respect to lesions in the small intestine, past studies in lambs, kids and calves infected with 10–100 times higher doses of oocysts than those in the present study reported focal haemorrhages and necrosis with ulcerations [9, 49, 50]; however, in our study, the small intestine was mildly affected, and inflammatory infiltrates were patchy, not correlated with parasite detection, and even found sporadically in uninfected animals.

Once infected by *T. gondii*, macrophages and neutrophils, as migratory leukocytes, rapidly spread to mesenteric lymph nodes, where the adaptive immune response is initiated. T cells are concentrated in the paracortical region and subcapsular sinus of the lymph nodes, which are important sites for T-cell priming, and can trigger effector functions such as cytokine release and cell killing to control pathogen spread in the host [22, 51–53]. On day 3 pi, adenomegaly of the mesenteric lymph nodes was rare, although follicular hyperplasia was observed in some animals, as previously described at this time point after infection in lambs and calves with *T. gondii* oocysts [9, 49]. At this time point, the detection of *T. gondii* DNA in the mesenteric lymph nodes was rare, although it highlights the parasite’s early entry into the lymphatic system in sheep, similar to previous reports [4]. Three days later, on day 6 pi, adenomegaly was found in all the jejunal lymph nodes as previously reported in sheep infected with sporulated oocysts [9]. At this time point, the number of T cells in the mesenteric lymph nodes is greater because of the stimulation of cell division [54]. Indeed, follicular hyperplasia in the cortex and sinus histiocytosis in the medulla were more widespread than on day 3 pi, as previously described [9]. In the same way, there was a significant increase in parasite burden in the jejunal mesenteric lymph nodes between day 3 and 6 pi, similar to previous reports [4]. Similarly, [46] reported greater *T. gondii* burdens in the PP and mesenteric lymph nodes than in the small intestine parenchyma. Parasite DNA detection and burden in the medial and distal jejunal lymph nodes were greater than those in other lymph nodes draining the small intestine, which, combined with the results in the small intestine, confirms these areas as predilection sites in sheep for *T. gondii* entry into the host after oral infection with oocysts. Three weeks later, on day 28 pi, no macroscopic lesions were observed, similar to previous reports in mice [55]. Microscopically, granulomatous lesions were found, in accordance with previous studies where these lesions were described in the mesenteric lymph nodes at three weeks after infection of lambs with *T. gondii* oocysts [56]. At this time point, the parasite DNA is still detected in the mesenteric lymph nodes [4, 5], although parasite burdens were lower compared to day 6 pi.

Dissemination through the bloodstream allows *T. gondii* to reach secondary tissues. The hijacking of macrophages and T cells by *T. gondii* type II isolates is a major route of parasite dissemination [53, 57–59]. Hence, we assessed the presence of parasite DNA in white blood cells, and it was incidental, with only one positive sample on day 10 pi, similar to previous studies in sheep where the parasite or parasite DNA was only sporadically detected in blood [60–62] or not detected [9].

At the placental level, at the time of early abortion (7–14 dpi), the presence of the parasite is very rare, but placental infarcts [16] and an increased presence of macrophages are observed [63]. In this study, at the time of early abortion, placental infarcts could not be evaluated because of autolysis during abortion. Prior to early abortions, on day 6 pi, no gross lesions in the placentomes were observed, similarly to a previous study in which placentomes on day 5 pi were evaluated [16]. However, under microscopic evaluation, at day 6 pi endothelial damage in placental vessels with extravasation of proteinaceous fluid and even formation of thrombi were observed in some placentomes. Taken together, these data suggest that placental thrombosis is a very acute process occurring from day 6 to 8 pi, when foetal death begins.

In early aborted foetuses, hypoxia caused by placental thrombosis triggered leukomalacia lesions in the brain as previously described [8, 16, 18, 32]. The occurrence of this lesion was confirmed by βAPP immunolabelled areas in the brains of foetuses aborted on day 8 pi., similar to previous reports [32]. Leukomalacia was not observed in brain foetuses euthanized on days 3 and 6 pi, but areas βAPP with cytoplasmic labelling were sporadically observed. The labelled cells included neurons, microglia, astrocytes and Purkinje cells. This type of cytoplasmic labelling may indicate an early stage of βAPP build-up in foetal brain cells, which is massively mobilised to the area of leukomalacia over a short span of time between days 6 and 8 pi; hence, it is tempting to hypothesize that in *T. gondii* induced early abortions, leukomalacia occurs in a hyperacute fashion shortly before foetal death. Since placental vascular lesions were first detected on day 6 pi, it would be expected that greater βAPP staining would be present in the brains of foetuses on day 6 than on day 3 pi. However, cytoplasmic staining was observed on both days 3 and 6 pi at similarly low levels. Hence, further studies are needed to characterize the βAPP staining patterns observed in foetal brains early after infection before abortion occurs. As expected, parasite DNA was not detected in placental or foetal tissues from early abortions or from sheep euthanized on days 3 and 6 pi but was widely detected in infected and non-aborted sheep on day 28 pi, as previously described [5, 19].

In conclusion, our results suggest that Peyer’s patches in the medial jejunum and the medial and distal jejunal mesenteric lymph nodes are key sites for the early replication and dissemination of *T. gondii* in sheep and for the initiation of immune responses. Interestingly, as soon as 6 days pi, microscopic endothelial activation and thrombotic lesions are observed in some placentomes, culminating in the appearance of early foetal death two days later. Further studies focusing on the target tissues of early *T. gondii* replication are necessary to delve deeper into the pathogenic mechanism of early abortion.

## Supplementary Information

**Additional file 1. Histopathology and parasite DNA detection and quantification in the small intestine**.

**Additional file 2. Macroscopic lesions, histopathology and parasite DNA detection in lymph nodes draining the small intestine**.

**Additional file 3. Histological lesions in the placentomes**.

**Additional file 4.** **βAPP immunolabelling in foetal brains.** For each foetal brain, 4 different areas were studied.

**Additional file 5. βAPP staining of foetal brains. **Representative images of the two different labelling patterns observed: i) staining of areas of variable size in the white matter (A, foetus from sheep 3.1) and ii) sparse areas where the cytoplasm of different cell types in foetal brains from infected sheep was labelled (B, D and F, corresponding to foetuses from sheep 3.4, 1.2 and 1.1, respectively), as opposed to those from the uninfected controls (C, E and G, corresponding to foetuses from sheep 5.2, 4.3 and 5.3, respectively). Neurons (B–C), microglia (D–E), and Purkinje cells in the cerebellum (F–G). Magnification: 40x (A) and 400x (B–G).
