# Supplementary material for: Early dynamics of Toxoplasma gondii infection in sheep inoculated at mid-gestation with archetypal type II oocysts
Source: Vet Res. 2025 Jul 1;56:134. doi: 10.1186/s13567-025-01557-1 (PMC12218951; doi:10.1186/s13567-025-01557-1)
Supplement: Supplementary file 1 — Additional file 1. Histopathology and parasite DNA detection and quantification in the small intestine. [file 13567_2025_1557_MOESM1_ESM.docx]

**Additional file 1. Histopathology and parasite DNA detection and quantification in the small intestine**

| Group | Ewe ref. | Duodenum | | |  | Proximal jejunum | | | | | | |  | Medial jejunum | | | | | | |  | Distal jejunum | | | | | | |  | Ileum | | |  | Ileocecal valve | | | |
| --- | --- | --- | --- | --- | --- | --- | --- | --- | --- | --- | --- | --- | --- | --- | --- | --- | --- | --- | --- | --- | --- | --- | --- | --- | --- | --- | --- | --- | --- | --- | --- | --- | --- | --- | --- | --- | --- |
|  |  |  |  |  |  | Wall | | |  | Wall + PP | | |  | Wall | | |  | Wall + PP | | |  | Wall | | |  | Wall +PP | | |  |  |  |  |  |  |  |  |  |
|  |  | HP | PCR | qPCR |  | HP | PCR | qPCR |  | HP | PCR | qPCR |  | HP | PCR | qPCR |  | HP | PCR | qPCR |  | HP | PCR | qPCR |  | HP | PCR | qPCR |  | HP | PCR | qPCR |  | HP | PCR | qPCR |  |
| Group 1  (infected, 3 dpi) | 1.1 |  |  |  |  |  |  |  |  | ++ |  |  |  |  |  |  |  | nr |  |  |  |  |  |  |  |  |  |  |  |  |  |  |  |  |  |  |  |
|  | 1.2 |  |  |  |  |  |  |  |  | ++ |  |  |  | ++ |  |  |  |  | 1 | 3.4 (1) |  |  |  |  |  |  |  |  |  |  |  |  |  | + |  |  |  |
|  | 1.3 |  |  |  |  |  | 1 |  |  |  | 1 |  |  | ++ | 1 |  |  |  |  |  |  |  |  |  |  | + |  |  |  | +++ |  |  |  |  |  |  |  |
|  | 1.4 |  |  |  |  |  |  |  |  | ++ |  |  |  |  |  |  |  |  |  |  |  |  |  |  |  | + |  |  |  | +++ |  |  |  |  |  |  |  |
|  | 1.5 |  |  |  |  | ++ |  |  |  |  |  |  |  | ++ |  |  |  |  |  |  |  |  |  |  |  |  |  |  |  |  |  |  |  |  |  |  |  |
| Group 2  (infected, 6 dpi) | 2.1 |  | 1 |  |  |  |  |  |  | ++ |  |  |  | ++ |  |  |  | ++ | 1 | 31.2 (1) |  | ++ |  |  |  |  |  |  |  |  |  |  |  |  |  |  |  |
|  | 2.2 |  |  |  |  |  |  |  |  |  |  |  |  |  |  |  |  |  |  |  |  | ++ | 1 |  |  | ++ |  |  |  |  |  |  |  | nr |  |  |  |
|  | 2.3 | ++ |  |  |  |  |  |  |  | ++ |  |  |  |  |  |  |  | nr | 2 | 9.5 (1) |  |  | 2 |  |  | ++ |  |  |  |  |  |  |  |  |  |  |  |
|  | 2.4 |  |  |  |  |  |  |  |  | nr |  |  |  | ++ |  |  |  | ++ | 3 | 69.5 ± 78.5 (2) |  | + |  |  |  | + |  |  |  | + | 1 | 2.9 (1) |  |  |  |  |  |
|  | 2.5 | ++ |  |  |  | ++ |  |  |  | nr |  |  |  | ++ |  |  |  | ++ |  |  |  | nr |  |  |  | ++ |  |  |  |  |  |  |  | +++ |  |  |  |
| Group 3 (infected, 28 dpi) | 3.1 |  |  |  |  | ++ |  |  |  | ++ |  |  |  | + | 1 |  |  | ++ | 2 |  |  |  |  |  |  |  | 2 |  |  |  |  |  |  |  | 1 |  |  |
|  | 3.2 |  |  |  |  |  |  |  |  |  |  |  |  | ++ | 1 |  |  | ++ |  |  |  |  |  |  |  |  |  |  |  |  |  |  |  |  |  |  |  |
|  | 3.3 |  |  |  |  | ++ |  |  |  | nr |  |  |  | ++ | 1 |  |  |  | 3 | 3.4 ± 0.6 (3) |  |  | 1 |  |  |  | 3 | 36.1 (1) |  |  |  |  |  | nr | 2 | 2.4 (1) |  |
|  | 3.4 |  |  |  |  |  |  |  |  | ++ | 1 |  |  | + |  |  |  |  |  |  |  |  |  |  |  |  |  |  |  |  |  |  |  |  |  |  |  |
|  | 3.5 | ++ |  |  |  |  |  |  |  |  |  |  |  | ++ |  |  |  |  |  |  |  |  |  |  |  |  |  |  |  |  |  |  |  |  |  |  |  |
| Group 4 (non-infected, 4 dpi) | 4.1 |  |  |  |  |  |  |  |  | ++ |  |  |  |  |  |  |  |  |  |  |  |  |  |  |  |  |  |  |  |  |  |  |  |  |  |  |  |
|  | 4.2 |  |  |  |  |  |  |  |  |  |  |  |  |  |  |  |  |  |  |  |  | + |  |  |  |  |  |  |  | ++ |  |  |  |  |  |  |  |
|  | 4.3 |  |  |  |  |  |  |  |  |  |  |  |  |  |  |  |  |  |  |  |  | + |  |  |  |  |  |  |  |  |  |  |  |  |  |  |  |
| Group 5 (non-infected, 28 dpi) | 5.1 |  |  |  |  | ++ |  |  |  | nr |  |  |  | ++ |  |  |  | nr |  |  |  |  |  |  |  |  |  |  |  |  |  |  |  |  |  |  |  |
|  | 5.2 | ++ |  |  |  |  |  |  |  | ++ |  |  |  | ++ |  |  |  | ++ |  |  |  |  |  |  |  |  |  |  |  |  |  |  |  |  |  |  |  |
|  | 5.3 |  |  |  |  |  |  |  |  |  |  |  |  |  |  |  |  |  |  |  |  |  |  |  |  | +++ |  |  |  |  |  |  |  |  |  |  |  |

PP: Peyer’s patches; HP: histopathological lesions; “+” histological changes without pathological significance such as congestion; “++” non-specific lesions including mild non-suppurative inflammatory infiltrates of the epithelium and lamina propria as well as intraepithelial follicles or reactive follicles in Peyer´s patches (in bold when at least 50% of the animals exhibited these lesions); “+++” lesions consistent with *T. gondii* infection such as granulomatous enteritis; nr: not representative samples. PCR: “1” parasite DNA detection in 1 out of 3 replicates of DNA extraction and PCR of each tissue; “2” parasite DNA detection in 2 out of 3 replicates of DNA extraction and PCR of each tissue; “3” parasite DNA detection in 3 out of 3 replicates of DNA extraction and PCR of each tissue. qPCR: mean ± standard deviation of the tachyzoites per mg of tissue; between brackets it is stated the number of nested-PCR positive samples in which quantification was possible.
