# Supplementary material for: Early dynamics of Toxoplasma gondii infection in sheep inoculated at mid-gestation with archetypal type II oocysts
Source: Vet Res. 2025 Jul 1;56:134. doi: 10.1186/s13567-025-01557-1 (PMC12218951; doi:10.1186/s13567-025-01557-1)
Supplement: Supplementary file 2 — Additional file 2. Macroscopic lesions, histopathology and parasite DNA detection in lymph nodes draining the small intestine. [file 13567_2025_1557_MOESM2_ESM.docx]

**Additional file 2. Macroscopic lesions, histopathology and parasite DNA detection in lymph nodes draining the small intestine**

| **Group** | **Ewe ref.** | **Proximal jejunal LN** | | | |  | **Medial jejunal LN** | | | |  | **Distal jejunal LN** | | | |  | **Terminal jejunal LN** | | | |  | **Ileocolic LN** | | | | |
| --- | --- | --- | --- | --- | --- | --- | --- | --- | --- | --- | --- | --- | --- | --- | --- | --- | --- | --- | --- | --- | --- | --- | --- | --- | --- | --- |
|  |  | **LES** | **HP** | **PCR** | **qPCR** |  | **LES** | **HP** | **PCR** | **qPCR** |  | **LES** | **HP** | **PCR** | **qPCR** |  | **LES** | **HP** | **PCR** | **qPCR** |  | **LES** | **HP** | **PCR** | **qPCR** |  |
| Group 1  (infected, 3 dpi) | 1.1 |  |  |  |  |  |  | ++ | 1 |  |  |  | ++ |  |  |  |  | + |  |  |  |  | ++ |  |  |  |
|  | 1.2 |  |  |  |  |  |  |  | 1 |  |  |  |  |  |  |  |  |  |  |  |  |  | ++ |  |  |  |
|  | 1.3 |  | ++ |  |  |  | LN |  | 2 |  |  | LN | ++ |  |  |  |  | ++ |  |  |  |  | ++ | 1 |  |  |
|  | 1.4 |  | ++ | 1 |  |  |  |  |  |  |  |  |  | 2 |  |  |  |  |  |  |  |  | ++ |  |  |  |
|  | 1.5 |  |  |  |  |  |  |  |  |  |  |  |  |  |  |  |  | ++ |  |  |  |  | ++ |  |  |  |
| Group 2  (infected, 6 dpi) | 2.1 | LN | ++ | 1 |  |  | LN | ++ | 2* | 10.6 ± 8.6 (2) |  | LN | ++ | 2 | 6.9 (1) |  | LN |  |  |  |  |  |  |  |  |  |
|  | 2.2 | LN | ++ | 3 | 9.7 ± 2.3 (3) |  | LN | ++ | 3 | 5.4 (1) |  | LN | ++ | 3 | 7 ± 1.4 (3) |  | LN | + | 2* | 28.9 ± 2.9 (2) |  |  |  |  |  |  |
|  | 2.3 | LN |  | 1** | 5.5 (1) |  | LN | + | 1** | 95.4 (1) |  | LN | + | 3 | 65 ± 21.7 (3) |  | LN | ++ | 3 | 10.5 ± 1.7 (2) |  |  | ++ |  |  |  |
|  | 2.4 | LN | + |  |  |  | LN | + | 3 | 37.5 ± 24.3 (3) |  | LN | ++ | 3 | 30.3 ± 13.7 (3) |  | LN | + | 2 |  |  | LN | ++ |  |  |  |
|  | 2.5 | LN |  |  |  |  | LN | ++ | 3 | 18.3 ± 4.7 (2) |  | LN | ++ | 3 | 7 ± 6.8 (3) |  | LN |  | 3 | 17.2 (1) |  |  | ++ |  |  |  |
| Group 3 (infected, 28 dpi) | 3.1 |  | ++ |  |  |  |  | ++ | 3 | 3.7 (1) |  |  |  | 2* |  |  |  |  | 2 |  |  |  | ++ | 2 |  |  |
|  | 3.2 |  |  | 2 |  |  |  | +++ |  |  |  |  |  | 2 |  |  |  |  |  |  |  |  |  |  |  |  |
|  | 3.3 |  |  | 2 | 6.7 (1) |  |  | +++ | 3 | 14.1 ± 6.9 (2) |  |  | +++ | 3 | 9.4 (1) |  |  |  | 3 | 5.4 (1) |  |  | ++ | 2* |  |  |
|  | 3.4 |  | +++ |  |  |  |  |  | 3 |  |  |  |  | 1 |  |  |  |  |  |  |  |  | ++ | 1 |  |  |
|  | 3.5 |  |  |  |  |  |  |  | 1 | 2.1 (1) |  |  |  | 2* |  |  |  | ++ | 3 |  |  |  | ++ |  |  |  |
| Group 4 (non-infected, 4 dpi) | 4.1 |  |  |  |  |  |  |  |  |  |  |  |  |  |  |  |  |  |  |  |  |  |  |  |  |  |
|  | 4.2 |  |  |  |  |  |  |  |  |  |  |  |  |  |  |  |  |  |  |  |  |  |  |  |  |  |
|  | 4.3 |  |  |  |  |  |  |  |  |  |  |  |  |  |  |  |  |  |  |  |  |  |  |  |  |  |
| Group 5 (non-infected, 28 dpi) | 5.1 | LN | ++ |  |  |  | LN | ++ |  |  |  | LN |  |  |  |  |  | ++ |  |  |  |  | ++ |  |  |  |
|  | 5.2 |  |  |  |  |  |  |  |  |  |  |  |  |  |  |  |  |  |  |  |  |  |  |  |  |  |
|  | 5.3 |  |  |  |  |  |  |  |  |  |  |  | ++ |  |  |  |  |  |  |  |  |  |  |  |  |  |

LES: macroscopic lesions; LN: lymphadenomegaly.
HP: histopathological lesions; “+” histological changes without pathological significance, such as congestion; “++” non-specific lesions, such as reactions in the cortical and/or medullary regions; “+++” lesions compatible with *T. gondii* infection, such as granulomas. PCR: “1” parasite DNA detection in 1 out of 3 replicates of DNA extraction and PCR of each tissue; “2” parasite DNA detection in 2 out of 3 replicates of DNA extraction and PCR of each tissue; “3” parasite DNA detection in 3 out of 3 replicates of DNA extraction and PCR of each tissue. * Insufficient sample for one replicate of the DNA extraction and PCR. ** Insufficient sample for two replicates of the DNA extraction and PCR. . qPCR: mean ± standard deviation of the tachyzoites per mg of tissue; between brackets it is stated the number of nested-PCR positive samples in which quantification was possible.
