# Supplementary material for: Early dynamics of Toxoplasma gondii infection in sheep inoculated at mid-gestation with archetypal type II oocysts
Source: Vet Res. 2025 Jul 1;56:134. doi: 10.1186/s13567-025-01557-1 (PMC12218951; doi:10.1186/s13567-025-01557-1)
Supplement: Supplementary file 3 — Additional file 3. Histological lesions in the placentomes. [file 13567_2025_1557_MOESM3_ESM.docx]

**Additional file 3. Histological lesions in the placentomes**

| **Group** | **Ewe ref.** | **Sequential culling (dpi)/ abortion (dpi)** | **Placentomes from the cranial region of the uterine horns** | | | | |  | | **Placentomes from the intermediate region of the uterine horns** | | | |  | | **Placentomes from the caudal region of the uterine horns** | | | | |  |  |
| --- | --- | --- | --- | --- | --- | --- | --- | --- | --- | --- | --- | --- | --- | --- | --- | --- | --- | --- | --- | --- | --- | --- |
|  |  |  | **PL1** | **PL2** | **PL3** | **PL4** |  | | **PL5** | | **PL6** | **PL7** | **PL8** | |  | **PL9** | **PL10** | **PL11** | | **PL12** | | |
| Group 1 (infected, 3 dpi) | 1.1 | SC (3) |  |  |  |  |  | | nr | | nr | nr | nr | |  |  |  |  | |  | | |
|  | 1.2 | SC (3) |  | nr | ++ |  |  | |  | | nr |  |  | |  | + | nr |  | | + | | |
|  | 1.3 | SC (3) |  | + | + |  |  | | + | | nr |  | nr | |  |  |  |  | |  | | |
|  | 1.4 | SC (3) |  | + | + | nr |  | | + | |  |  |  | |  |  |  |  | |  | | |
|  | 1.5 | SC (3) |  |  | ++ | nr |  | |  | |  |  | + | |  |  |  |  | |  | | |
| Group 2 (infected, 6 dpi) | 2.1 | SC (6) | + | nr | + |  |  | | + | | + | + | + | |  | + | + |  | | ++ | | |
|  | 2.2 | SC (6) | ++ | ++ | ++ |  |  | |  | | ++ |  | + | |  | +++ | + | +++ | | +++ | | |
|  | 2.3 | SC (6) |  | ++ | ++ | + |  | |  | |  |  | ++ | |  |  | ++ | +++ | |  | | |
|  | 2.4 | SC (6) |  |  |  |  |  | | + | |  |  |  | |  |  |  |  | |  | | |
|  | 2.5 | SC (6) |  | ++ |  | ++ |  | |  | |  |  |  | |  |  |  |  | |  | | |
| Group 3 (infected, 28 dpi) | 3.1 | A (8)/SC (28) | NA | NA | NA | NA |  | | NA | | NA | NA | NA | |  | NA | NA | NA | | NA | | |
|  | 3.2 | A (8)/SC (28) | NA | NA | NA | NA |  | | NA | | NA | NA | NA | |  | NA | NA | NA | | NA | | |
|  | 3.3 | A (8)/SC (28) | NA | NA | NA | NA |  | | NA | | NA | NA | NA | |  | NA | NA | NA | | NA | | |
|  | 3.4 | SC (28) | ++ | + |  | ++ |  | | + | |  |  | + | |  |  |  | + | | + | | |
|  | 3.5 | A (8)/SC (28) | NA | NA | NA | NA |  | | NA | | NA | NA | NA | |  | NA | NA | NA | | NA | | |
| Group 4 (non-infected, 4 dpi) | 4.1 | SC (4) | + |  | + | + |  | |  | | + | + |  | |  | + |  | + | | + | | |
|  | 4.2 | SC (4) |  |  |  | + |  | | + | | + |  | + | |  | + |  | + | | + | | |
|  | 4.3 | SC (4) | + | + | ++ | ++ |  | | ++ | |  | + | ++ | |  |  |  |  | | ++ | | |
| Group 5 (non-infected, 28 dpi) | 5.1 | SC (28) |  |  |  |  |  | |  | |  |  |  | |  |  |  |  |  | | |  |
|  | 5.2 | SC (28) | + | + | + | + |  | |  | |  |  | + | |  |  | + | + | + | | |  |
|  | 5.3 | SC (28) |  |  |  |  |  | |  | |  |  |  | |  | ++ | + | + |  | | |  |

PL: placentome: SC: sequential culling; A: abortion; NA: not collected for histopathology; nr: not representative samples; Histopathological score, “+” mild histological changes without clear pathological relevance including findings such as calcifications or the accumulation of cellular debris between villi; “++” non-specific histological changes, including features such as vascular congestion, endothelial activation, intravascular coagulation, or pyknosis of the maternal epithelium; “+++” vascular lesions with greater pathological significance, including lesions of the vascular walls, hemorrhages and thrombi.
