# Supplementary material for: Early dynamics of Toxoplasma gondii infection in sheep inoculated at mid-gestation with archetypal type II oocysts
Source: Vet Res. 2025 Jul 1;56:134. doi: 10.1186/s13567-025-01557-1 (PMC12218951; doi:10.1186/s13567-025-01557-1)
Supplement: Supplementary file 4 — Additional file 4. βAPP immunolabelling in foetal brains. For each foetal brain, 4 different areas were studied. [file 13567_2025_1557_MOESM4_ESM.docx]

**Additional file 4. βAPP-immunolabelling in foetal brains.** For each foetal brain, 4 different areas were studied.

| **Group** | **Ewe ref.** | **Foetus ref.** | **Sequential culling (dpi)/ abortion (dpi)** | **Frontal lobe (Slide A)** | **Corpus Callosum (Slide B)** | **Midbrain (Slide C)** | **Cerebellum (Slide D)** |
| --- | --- | --- | --- | --- | --- | --- | --- |
|  |  |  |  |  |  |  |  |
| **Group 1**  **(infected, 3 dpi)** | 1.1 | 1.1 F1 | SC (3) |  |  | ++ neurons | + Purkinje |
|  |  | 1.1 F2 | SC (3) |  |  | + microglia and neurons |  |
|  | 1.2 | 1.2 F1 | SC (3) |  |  |  |  |
|  |  | 1.2 F2 | SC (3) |  | + microglia | + neurons |  |
|  | 1.3 | 1.3 F1 | SC (3) |  |  |  |  |
|  |  | 1.3 F2 | SC (3) | + microglia | + microglia | ++ neurons |  |
|  | 1.4 | 1.4 F1 | SC (3) |  |  |  |  |
|  |  | 1.4 F2 | SC (3) |  |  |  |  |
|  | 1.5 | 1.5 F1 | SC (3) |  |  |  |  |
|  |  | 1.5 F2 | SC (3) |  |  |  |  |
| **Group 2**  **(infected, 6 dpi)** | 2.1 | 2.1 F1 | SC (6) |  |  |  |  |
|  | 2.2 | 2.2 F1 | SC (6) |  |  |  | ++ Purkinje and neurons |
|  |  | 2.2 F2 | SC (6) | + microglia and neurons |  |  |  |
|  |  | 2.2 F3 | SC (6) |  |  |  |  |
|  | 2.3 | 2.3 F1 | SC (6) |  |  | + neurons |  |
|  | 2.4 | 2.4 F1 | SC (6) | + microglia |  |  |  |
|  | 2.5 | 2.5 F1 | SC (6) |  |  |  | + Purkinje and neurons |
|  |  | 2.5 F2 | SC (6) |  |  |  |  |
| **Group 3 (infected, 28 dpi)** | 3.1 | 3.1 F1 | SC (28) / A (8) | +++ AREA | ++ AREA | ++ AREA and astrocytes |  |
|  |  | 3.1 F2 | SC (28) / A (8) | + AREA and astrocytes |  | + AREA |  |
|  | 3.2 | 3.2 F1 | SC (28) / A (8) | + AREA | + AREA | + AREA |  |
|  |  | 3.2 F2 | SC (28) / A (8) |  | + AREA |  |  |
|  |  | 3.2 F3 | SC (28) / A (8) |  |  |  |  |
|  | 3.3 | 3.3 F1 | SC (28) / A (8) | +++ AREA |  |  |  |
|  |  | 3.3 F2 | SC (28) / A (8) | +++ AREA | ++ AREA |  |  |
|  |  | 3.3 F3 | SC (28) / A (8) | ++ AREA |  |  |  |
|  | 3.4 | 3.4 F1 | SC (28) | + astrocytes, microglia |  |  | + neurons and Purkinje |
|  |  | 3.4 F2 | SC (28) |  |  | + microglia | + neurons |
|  |  | 3.4 F3 | SC (28) |  |  |  | + neurons |
|  | 3.5 | 3.5 F1 | SC (28) / A (8) |  |  |  |  |
|  |  | 3.5 F2 | SC (28) / A (8) | ++ AREA and neurons | ++ AREA |  |  |
|  |  | 3.5 F3 | SC (28) / A (8) | ++ AREA | + AREA and astrocytes | +++ AREA |  |
| **Group 4 (non-infected, 4 dpi)** | 4.1 | 4.1 F1 | SC (4) |  |  |  |  |
|  |  | 4.1 F2 | SC (4) |  |  |  |  |
|  |  | 4.1 F3 | SC (4) |  |  |  |  |
|  |  | 4.1 F4 | SC (4) |  |  |  |  |
|  | 4.2 | 4.2 F1 | SC (4) |  |  |  |  |
|  | 4.3 | 4.3 F1 | SC (4) |  |  |  |  |
|  |  | 4.3 F2 | SC (4) |  |  |  |  |
| **Group 5 (non-infected, 28 dpi)** | 5.1 | 5.1 F1 | SC (28) |  |  |  |  |
|  |  | 5.1 F2 | SC (28) |  |  |  |  |
|  | 5.2 | 5.2 F1 | SC (28) |  |  |  |  |
|  |  | 5.2 F2 | SC (28) |  |  |  |  |
|  | 5.3 | 5.3 F1 | SC (28) |  |  |  |  |
|  |  | 5.3 F2 | SC (28) |  |  |  |  |

“+” indicates sporadic or focal staining, “++” focal, but widely distributed staining, and “+++” generalized spread staining. F: Foetus, SC: Sequential culling, A: abortion. AREA: indicates classical leukomalacia foci staining. When specific cell types are indicated, the staining is restricted to the cytoplasm of those cell types.
