# Supplementary figures and images for: Early dynamics of Toxoplasma gondii infection in sheep inoculated at mid-gestation with archetypal type II oocysts
Source: Vet Res. 2025 Jul 1;56:134. doi: 10.1186/s13567-025-01557-1 (PMC12218951; doi:10.1186/s13567-025-01557-1)

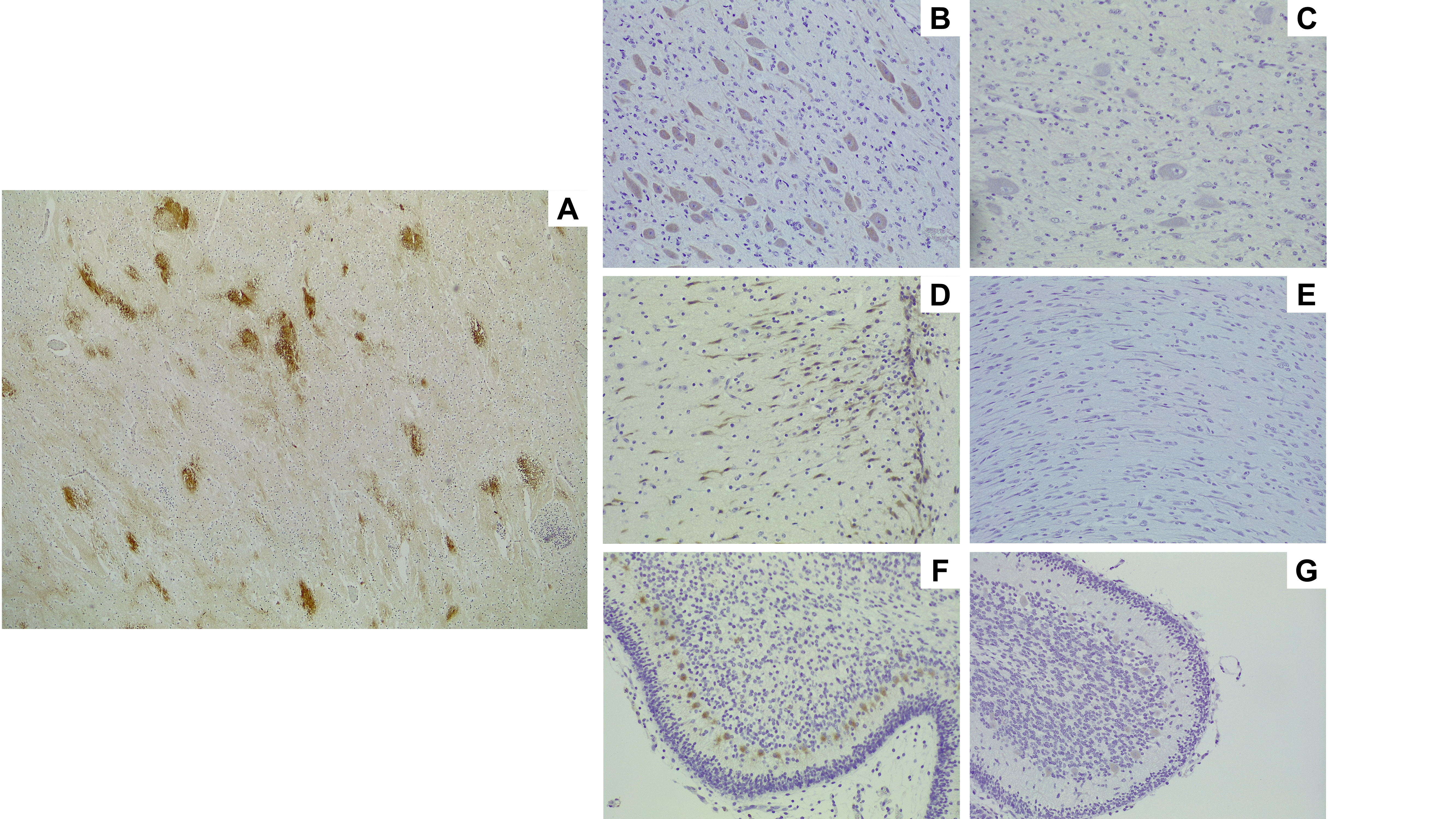

Supplement: Supplementary file 5 — Additional file 5. βAPP staining of foetal brains. Representative images of the two different labelling patterns observed: i) staining of areas of variable size in the white matter (A, foetus from sheep 3.1) and ii) sparse areas where the cytoplasm of different cell types in foetal brains from infected sheep was labelled (B, D and F, corresponding to foetuses from sheep 3.4, 1.2 and 1.1, respectively), as opposed to those from the uninfected controls (C, E and G, corresponding to foetuses from sheep 5.2, 4.3 and 5.3, respectively). Neurons (B–C), microglia (D–E), and Purkinje cells in the cerebellum (F–G). Magnification: 40x (A) and 400x (B–G). [file 13567_2025_1557_MOESM5_ESM.tif]
